# Lack of association between pandemic chilblains and SARS-CoV-2 infection

**DOI:** 10.1073/pnas.2122090119

**Published:** 2022-02-25

**Authors:** Jeff R. Gehlhausen, Alicia J. Little, Christine J. Ko, Marc Emmenegger, Carolina Lucas, Patrick Wong, Jon Klein, Peiwen Lu, Tianyang Mao, Jillian Jaycox, Eric Wang, Yale IMPACT Team, Nelson Ugwu, Cate Muenker, Dilgash Mekael, Rhonda Q. Klein, Robert Patrignelli, Richard Antaya, Jennifer McNiff, William Damsky, Kathy Kamath, John Shon, Aaron M. Ring, Inci Yildirim, Saad Omer, Albert I. Ko, Adriano Aguzzi, Akiko Iwasaki

**Affiliations:** ^a^Yale Department of Dermatology, Yale University, New Haven, CT 06510;; ^b^Yale Department of Pathology, Yale University, New Haven, CT 06510;; ^c^Institute of Neuropathology, University Hospital of Zürich, 8091 Zürich, Switzerland;; ^d^Yale Department of Immunobiology, Yale University, New Haven, CT 06510;; ^e^Department of Epidemiology of Microbial Diseases, Yale School of Public Health, New Haven, CT 06510;; ^f^Modern Dermatology, Westport, CT 06880;; ^g^Patrignelli Dermatology Private Practice, Trumbull, CT 06611;; ^h^Serimmune, Inc., Santa Barbara, CA 93117;; ^i^Yale Institute for Global Health, Yale University, New Haven, CT 06510;; ^j^Department of Pediatrics, Section of Infectious Diseases, Yale University School of Medicine, New Haven, CT 06510;; ^k^Yale Department Medicine, Section of Infectious Diseases, Yale University, New Haven, CT 06510;; ^l^Department of Molecular Cellular and Developmental Biology, Yale University, New Haven, CT 06510;; ^m^HHMI, Chevy Chase, MD 20815

**Keywords:** chilblain, interferon, pernio, covid toe, SARS-CoV-2

## Abstract

An increased incidence of chilblains has been observed during the severe acute respiratory syndrome coronavirus 2 (SARS-CoV-2) pandemic and attributed to viral infection. Direct evidence of this relationship has been limited, however, as most cases do not have molecular evidence of prior SARS-CoV-2 infection with PCR or antibodies. We enrolled a cohort of 23 patients who were diagnosed and managed as having SARS-CoV-2–associated skin eruptions (including 21 pandemic chilblains [PC]) during the first wave of the pandemic in Connecticut. Antibody responses were determined through endpoint titration enzyme-linked immunosorbent assay and serum epitope repertoire analysis. T cell responses to SARS-CoV-2 were assessed by T cell receptor sequencing and in vitro SARS-CoV-2 antigen-specific peptide stimulation assays. Immunohistochemical and PCR studies of PC biopsies and tissue microarrays for evidence of SARS-CoV-2 were performed. Among patients diagnosed and managed as “covid toes” during the pandemic, we find a percentage of prior SARS-CoV-2 infection (9.5%) that approximates background seroprevalence (8.5%) at the time. Immunohistochemistry studies suggest that SARS-CoV-2 staining in PC biopsies may not be from SARS-CoV-2. Our results do not support SARS-CoV-2 as the causative agent of pandemic chilblains; however, our study does not exclude the possibility of SARS-CoV-2 seronegative abortive infections.

## Significance

Chilblain diagnoses have increased during the severe acute respiratory syndrome coronavirus 2 (SARS-CoV-2) pandemic and have been attributed to viral infection and a subsequent robust antiviral immune response. As a result, providers have managed these cases differently than idiopathic chilblains, which are associated with cold exposure. The relationship between pandemic chilblains and SARS-CoV-2 infection, however, remains unclear as most patients do not test positive for SARS-CoV-2–specific PCR or antibodies. To better understand this disconnect, we enrolled cases of pandemic chilblains in a study and performed detailed immune profiling of antibody and T cell responses. Additionally, we compared immunohistochemical staining of pandemic chilblains with prepandemic tissues. Our results do not support SARS-CoV-2 as the cause of the increased chilblain incidence.

Concurrent with the rise of COVID-19 cases worldwide during the pandemic in early 2020, reports from different groups on different continents described increased diagnoses of chilblains attributed to severe acute respiratory syndrome coronavirus 2 (SARS-CoV-2) infection (1–8). Chilblains are an acral inflammatory rash typically affecting the toes and fingers of adults in colder, wetter conditions without a known association with respiratory viruses. Arguments for an association between this entity titled “covid toe” (which we refer to as pandemic chilblains [PC]) and SARS-CoV-2 infection include clustering of chilblains in areas with high incidence of COVID-19, SARS-CoV-2 exposure/symptoms in a significant percentage of PC cases, positive staining of spike (S) antigen in some biopsies, increased incidence in warmer temperatures in spring/summer of 2020, an expanded body distribution and possibly more severe, recalcitrant type of chilblain eruption, and the absence of a history of chilblains and/or other laboratory associations with classic chilblains (9–11).

Despite this purported association between PC and SARS-CoV-2 infection, the majority of these patients lack evidence of prior infection (1, 4–8). Although testing was not widely available in early studies, this relationship has nonetheless held in later studies with more comprehensive testing (2, 3, 12). Arguments for this persistent inability to detect prior infection include 1) a missed window, with PCR testing too late and antibody testing too early; 2) loss of antibody positivity over time; and 3) that PC patients may feature a robust SARS-CoV-2 innate immune response that impedes the development of a detectable antibody signal (13). Thus, the association between PC and SARS-CoV-2 critically relies on the expectation that a significant number of these cases without evidence of prior infection did indeed experience infection that has not been successfully detected. We hypothesized that in-depth immunological profiling of both antibody and T cell responses of convalescent patients may resolve this question. Herein, we report our findings from a small cohort of PC patients that do not support an association between PC and prior SARS-CoV-2 infection.

## Results

### Clinical.

**Fig. 1.**
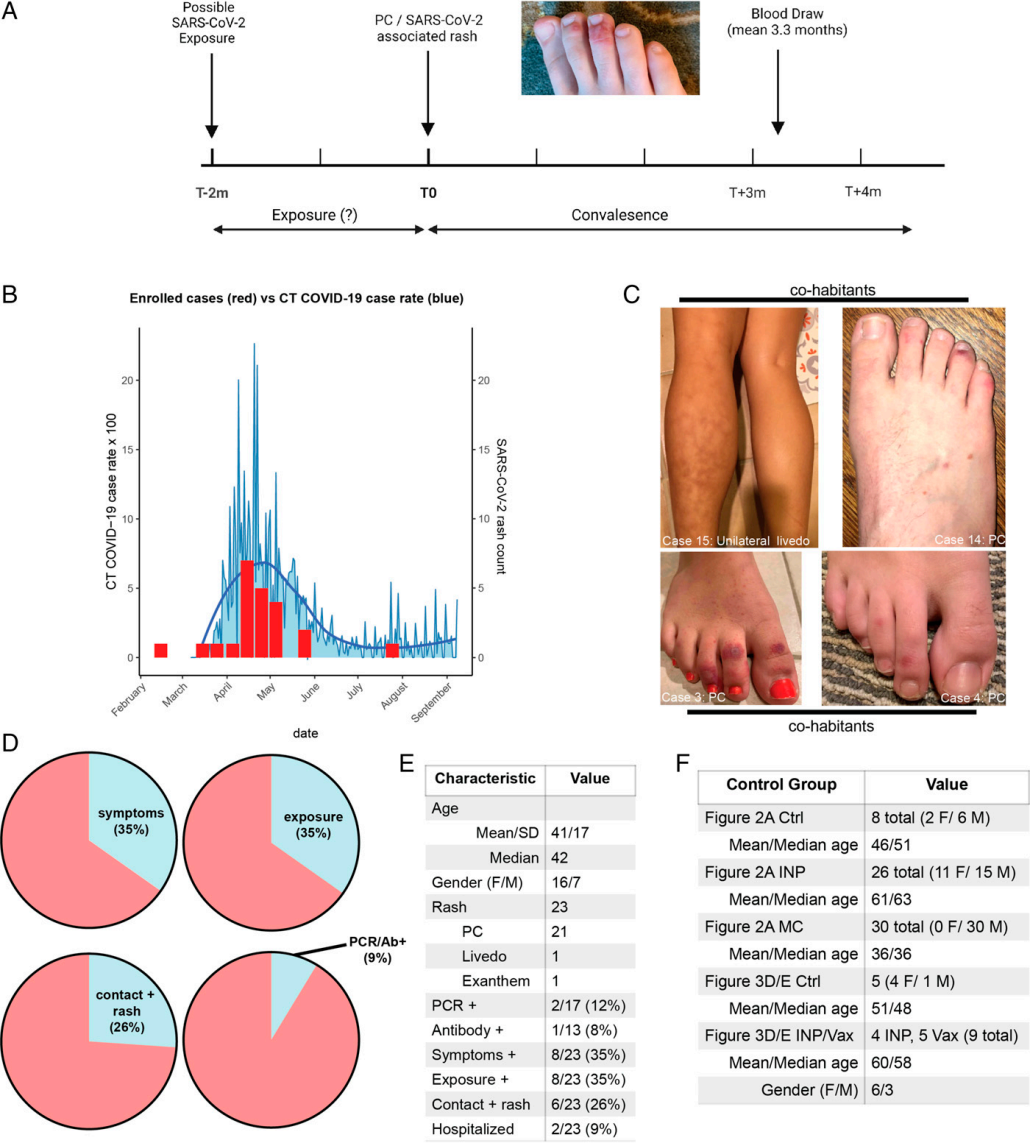
Clinical characteristics of the PC cohort. (*A*) Schema of our clinical study of the PC cohort (created in BioRender). Patients had a preceding potential exposure period of up to 2 mo; mean time to blood draw after onset of rash was 3.3 mo. All cases were resolved at the time of blood draw. (*B*) Histogram comparing case counts in the first wave of COVID-19 in 2020 in Connecticut (CT) to the timing of rashes in our cohort. (*C*) Clinical photos of two different households (four patients total) with SARS-CoV-2–associated rashes that developed in a concurrent time line. (*D*) Pie charts of clinical characteristics of the PC cohort, including symptoms, suspected or confirmed SARS-CoV-2 exposure, contacts with a rash attributed to SARS-CoV-2 infection (chilblains or livedo), and diagnostic testing results. (*E*) More detailed breakdown of demographics and clinical characteristics of the PC cohort. (*F*) Demographic and numeric data of control groups used in Figs. 2 and 3. Ab, antibody; Ctrl, control; F, female; M, male.

Twenty-three patients with a previous eruption attributed to SARS-CoV-2 during the first wave of the pandemic in 2020 were enrolled in our study (Fig. 1*A*). Patients with a prior history of chilblains or cutaneous lupus were excluded. Of these eruptions, 21 were chilblains, 1 was a viral exanthem, and 1 was unilateral livedo reticularis; this cohort is referred to collectively in the manuscript as PC unless otherwise noted. These different classes of eruptions all have a published association with SARS-CoV-2 infection (5). Chilblains have been described as a late finding, developing approximately 1 mo after suspected infection (6, 14). The mean duration of time between rash presentation and blood draw in our cohort was 3.3 mo. All rashes had resolved by the time of blood draw. Notably, the majority of rashes presented during a period of time in April and May 2020 coinciding with the peak local Connecticut infection rate, implying a temporal association between the spread of SARS-CoV-2 and cases in our cohort (Fig. 1*B*). Four cases included rashes from two separate households (two cases each) presenting with SARS-CoV-2–associated eruptions in an overlapping time line, suggesting a communicable cause within the household (Fig. 1*C*). Photographic evidence of all cases in the cohort is seen in *SI Appendix*, Fig. S1.

**Fig. 2.**
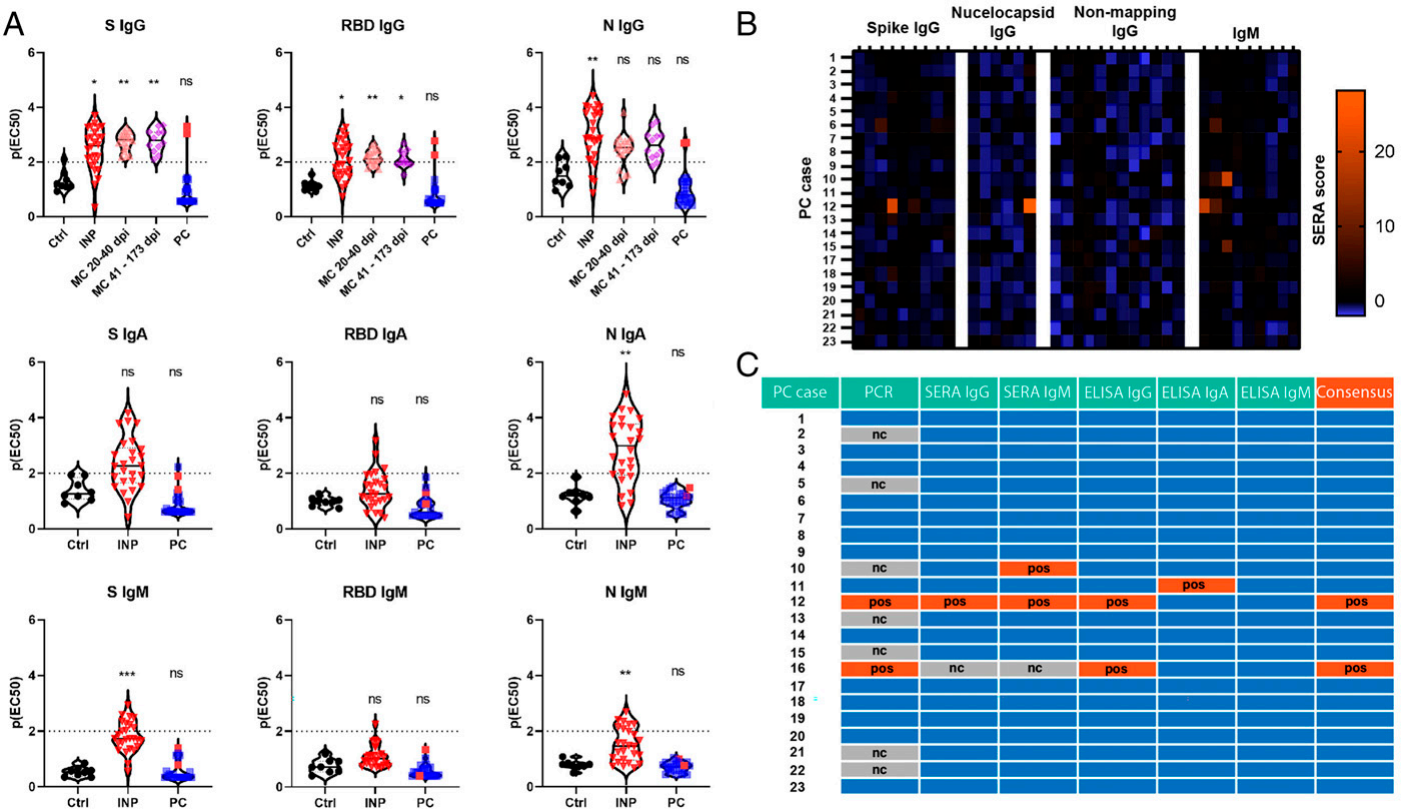
Convalescent antibody studies of PC cases compared with controls. (*A*) ELISA IgG, IgA, and IgM assays targeting S, RBD, and N antigens revealing antibody responses in indicated cohorts. The dotted lines at p(EC_50_) of two indicate the threshold for positivity. For details on p(EC50) please see *Methods*. Red dots in the PC group indicate previous confirmed positive cases (two total). (*B*) Heat map of SERA responses in the PC cohort for individual cases. Values at or above 25 are considered positive. (*C*) Consensus table summarizing positivity from various assays for each PC case. Blue boxes are negative. For all experiments with significance testing, Kruskal–Wallis test with post hoc Dunn’s test for multiple comparisons was employed. Ctrl, no exposure negative control; nc, not completed; ns, not significant; pos, positive. *P < 0.05; **P < 0.01; ***P < 0.001.

**Fig. 3.**
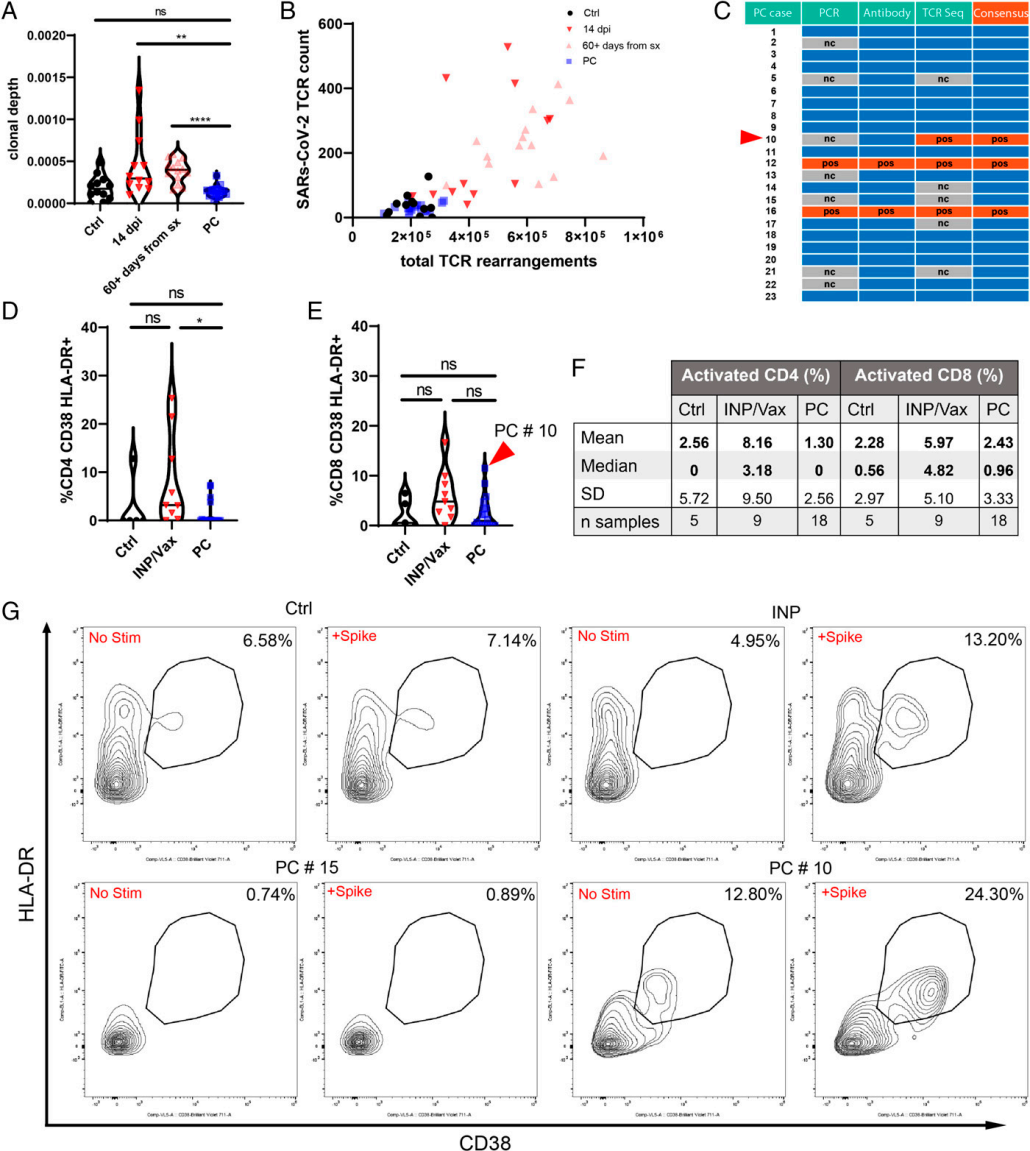
T cell studies of PC cases compared with controls. (*A*) Clonal depth of PC cases compared with indicated cohorts as assessed by TCR sequencing of the CD8 CDR3 region. Clonal depth is a ratio of the sum of antigen-specific (SARS-CoV-2) TCRs compared with the total productive TCR rearrangements. (*B*) Scatterplot relating the number of SARS-CoV-2–mapped TCRs to the number of rearranged TCRs per sample. For *A* and *B*, outliers that were more than three SDs from the median of each cohort were removed for visualization purposes: Ctrl (one), 14 dpi (two), 60+ d from symptoms (sx) (three), and PC (one). (*C*) Consensus table summarizing the results for antibody and T cell studies for PC cases. Blue boxes are negative. The red arrowhead denotes PC #10, which was not confirmed positive by antibody testing but was by TCR sequencing. (*D* and *E*) Violin plots of T cell responses for CD4 (*D*) and CD8 (*E*) responses to S-peptide stimulation. Percentages reflect the stimulated responses subtracting the baseline activation. PC #10 had the most robust CD8+ T cell activation in response to S in the PC cohort. (*F*) Descriptive statistics of S peptide stimulation studies. (*G*) Flow plots for representative CD8 T cell responses for Ctrl, INP/Vax, and PC cases in addition to PC #10, which was identified by TCR sequencing and peptide stimulation studies as having prior SARS-CoV-2 exposure. For all experiments with significance testing, Kruskal–Wallis test with post hoc Dunn’s test for multiple comparisons was employed. There were five INP and four Vax. Ctrl, no exposure negative control; nc, not completed; ns, not significant; pos, positive. *P < 0.05; **P < 0.01; ****P < 0.0001.

This cohort exhibited other characteristics supportive of prior SARS-CoV-2 infection. Thirty-five percent of patients had preceding symptoms they attributed to possible SARS-CoV-2 infection in the 2 mo prior to the eruption inclusive of fevers, cough, shortness of breath, and changes in smell or taste (Fig. 1*D*). Thirty-five percent reported exposure to either a confirmed or suspected case of COVID-19, while 26% of patients also reported a presumed SARS-CoV-2–associated eruption (chilblain or livedo) in a close contact prior to their own rash (Fig. 1*D*). Two cases (9%) had either a nasopharyngeal PCR or antibody test confirming infection, consistent with previous reports showing only a minority of PC cases with confirmed SARS-CoV-2 infection (Fig. 1*D*). Other characteristics of the cohort are seen in Fig. 1*E* and *SI Appendix*, Table S1. The characteristics of control groups used for the immunological studies in Figs. 2 and 3 are detailed Fig. 1*F* and Dataset S1. Of the 14 cases in our PC cohort where documentation of the first encounter with a health care professional was available, in 13 of these cases (93%) the favored diagnosis included PC caused by SARS-CoV-2, and/or initial management included sending a SARS-CoV-2 PCR test. These data demonstrate that our cohort of cases was diagnosed and managed as SARS-CoV-2–associated eruptions and additionally, well represents the published reports of PC.

### Serology.

We first analyzed the antibody responses of our cohort with endpoint titration enzyme-linked immunosorbent assay (ELISA) using S, receptor binding domain (RBD), and nucleocapsid (N) antigens for IgG, IgA, and IgM isotypes. For all antigens and isotypes, there was no difference between no exposure negative controls and PC (Fig. 2*A*). Positive controls included mixed severity inpatients (INPs) and a convalescent group with preceding asymptomatic or mild SARS-CoV-2 infections (MC) that did not result in hospitalization. Blood draw time points for the MC group extended to nearly 6 mo postinfection for some samples, and all remained seropositive. PCs #12 and #16, which had previous confirmed infections, were the only PC cases with positive antibodies. PC #11 had a borderline positive IgA signal, although lacked IgG, and thus, was not considered a confirmed positive.

We additionally completed serum epitope repertoire analysis (SERA) antibody profiling on our PC cohort (Fig. 2*B*). PC #12 was again identified as positive; PC #16 was not tested. Positive IgM signal was seen in PC #10 without IgG confirmation. Additional MC positive controls showed that these samples remained seropositive up to 1 y postinfection (*SI Appendix*, Table S2). In summary, antibody studies did not reveal any new evidence of infection in the PC cohort, and additionally, we demonstrate the ability of these assays to detect mild convalescent SARS-CoV-2 infections for 6 mo to a year afterward.

Functional autoantibodies were recently discovered to play a role in the severity of COVID-19 disease via unbiased rapid extracellular antigen profiling (REAP) technology (15). Additionally, antibody complexes have been observed in PC biopsies, suggesting the possibility of an antibody and/or immune complex–mediated pathology (16). We utilized REAP to assess the role of autoantibodies in PC. We found no differences between no exposure negative controls and PC (*SI Appendix*, Fig. S2 *A–D*). REAP RBD antigen testing did not identify any new SARS-CoV-2 antibodies in PC patients (*SI Appendix*, Fig. S2*E*).

### T Cell Immunity.

It has been hypothesized, but not shown, that a profound T cell response in PC patients may preclude the development of antibody responses and thus, explain the largely negative antibody results seen in published studies. To address this question, we utilized two orthogonal approaches to assay the T cell response in our cohort: complementary determining region 3 (CDR3) TCRβ sequencing of CD8 T cells using Adaptive Biotechnology’s immunoSEQ T-MAP COVID platform as well as S peptide in vitro stimulation assays to examine antigen-specific responses to SARS-CoV-2. T cell receptor (TCR) sequencing and peptide stimulation assays were performed on 18 samples each compared with negative and positive controls; 22 of 23 PC samples underwent at least one T cell assay.

TCR sequencing from frozen peripheral blood mononuclear cells (PBMCs) resolved over 100,000 productive TCR rearrangements for all sequenced samples (mean: 221,730; range: 107,993 to 313,888 templates). The immunoSEQ T-MAP COVID platform was utilized to map SARS-CoV-2 antigen-specific TCR templates present in the PC samples. As seen in Fig. 3 *A* and *B*, the clonal depth (sum of SARS-CoV-2 antigen-specific TCRs divided by the total productive TCR rearrangements) of the PC cohort was comparable with the negative control comparison group and statistically different from both 14-d postinfection (dpi) positive controls as well as a convalescent comparison group that was 60+ d postsymptom resolution (Dataset S2). The Adaptive Biotechnology COVID classifier (the same machine learning tool underlying their Food and Drug Administration-approved T-Detect testing platform) was employed on these samples as well. Of the 18 tested samples, 3 were positive, and the rest were negative. Two of these samples (PCs #12 and #16) had previously confirmed infections. The other positive sample was PC #10, which did have a positive IgM from the SERA assay (Fig. 3*C*).

In vitro T cell stimulation assays of PBMCs were performed utilizing a pool of S-protein peptides covering S1 and S2 subunits with 316 distinct overlapping 15-mer peptides. PC samples were compared against no exposure negative controls and a positive control group including COVID-19 inpatients and healthy vaccinated patients (INP/Vax). Flow cytometry analysis of CD4 and CD8+ HLA-DR CD38+ activated lymphocytes revealed comparable low signals in both the negative control and PC groups after S stimulation, whereas the INP/Vax group trended higher and was statistically different from the PC group in activated CD4+ cells (Fig. 3 *D–G*). Of the five PC samples that lacked TCR sequencing data, four of them had results from T cell stimulation assays. PCs #5, #14, #15, and #17 all had low or absent T cell responses in both CD4 and CD8 T cells, suggesting a lack of prior exposure to SARS-CoV-2. PC #10 was the only sample positive from the COVID classifier that also had data from T cell stimulation assays. Notably, this sample had the most robust CD8 T cell response of the PC cohort, with an 11.5% shift in response to S stimulation (Fig. 3 *E–G*), confirming the results from TCR sequencing.

### Pathology.

**Fig. 4.**
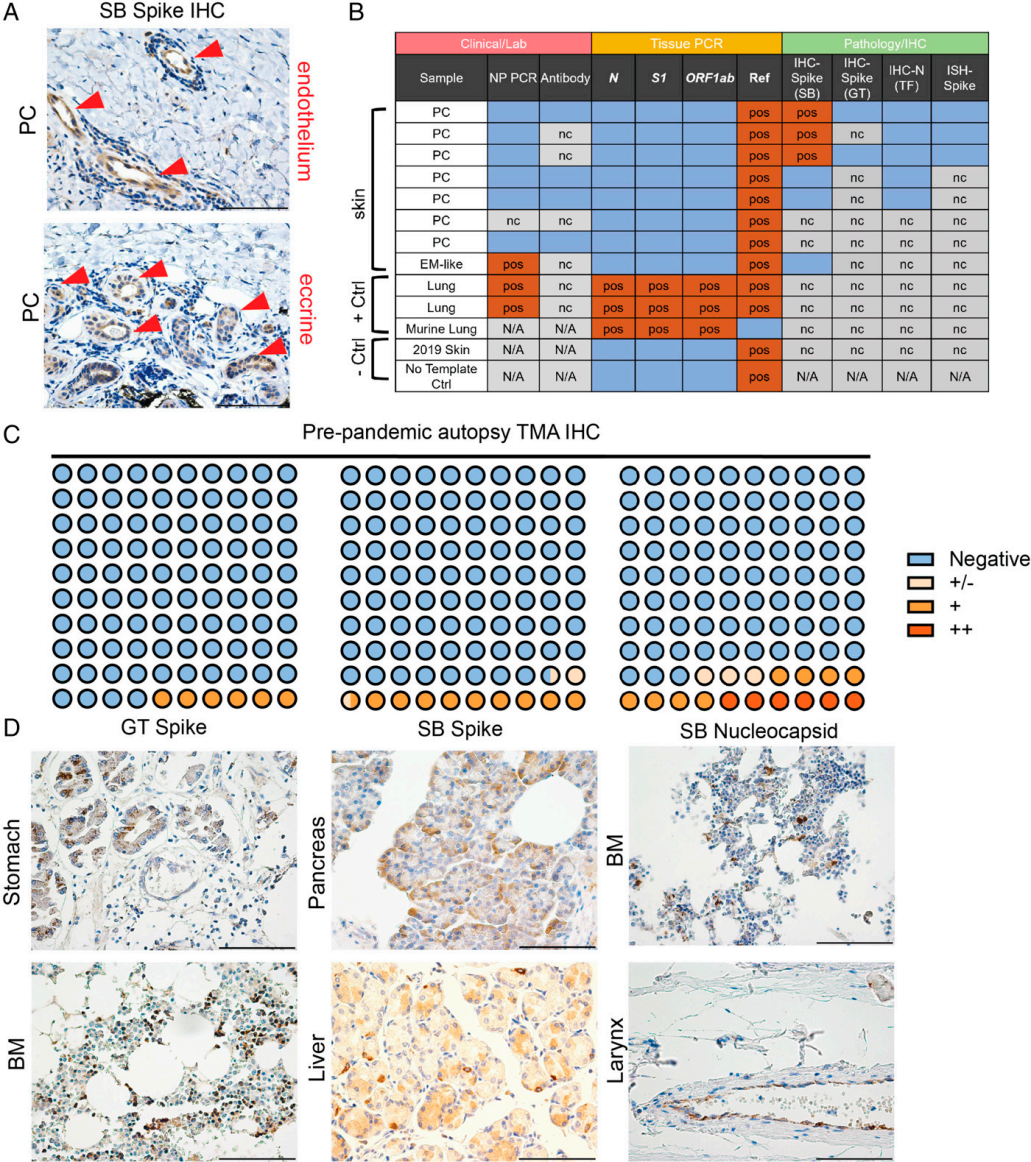
Immunohistochemical and PCR analyses of PC cases and TMAs. (*A*) Representative staining of the three positive PC cases of S IHC previously published by our group with focal staining in endothelium and eccrine glands (red arrowheads). (*B*) Summary of laboratory, PCR, and immunohistochemical analyses of SARS-CoV-2–associated skin rashes (chilblains and erythema multiforme [EM]–like lesions) compared with controls. Blue boxes are negative. (*C* and *D*) Dot plot summarizing TMA IHC results for respective antibodies. Representative staining for indicated antibodies is seen in *D*. *Methods* has a detailed discussion of antibodies and staining as well as PCR protocol. All images are 400× original magnification. BM, bone marrow; GT, GeneTex; N/A, not available; nc, not completed; NP, nasopharyngeal; pos, positive; SB, Sino Biologics; TF, ThermoFisher. (Scale bars: 100 μM.)

Some members of our group (J.R.G., C.J.K., J.M., and W.D.) previously published positive staining of S in the endothelium and eccrine glands of PC biopsies, which has been reported by other groups and used as supportive evidence of SARS-CoV-2 infection in PC cases (10, 17). In our case series, these three positive samples were negative for N-protein staining with immunohistochemistry (IHC) and for S messenger RNA with in situ hybridization (ISH) studies (Fig. 4*A* and *SI Appendix*, Fig. S4*A*). This led us to consider three possible conclusions: PC cases feature persistent S expression in the absence of RNA, S staining is a false positive, or the S antibody is reactive to a host antigen. To further examine this question, S staining was performed with a different antibody (GeneTex S) on two of the three cases, which were both negative. One of these two cases was included in our immunological analyses (PC #23) and had no evidence of prior infection in any study. RT-PCR from these and other PC or SARS-CoV-2 skin eruptions (*n* = 8) was also performed; all cases were negative for SARS-CoV-2 RNA (Fig. 4*B*).

To further explore the specificity of SARS-CoV-2–targeted antibodies, we performed IHC on prepandemic autopsy tissue microarrays (TMAs) from prior to 2019 that could not have potentially encountered SARS-CoV-2. In the case of all three tested antibodies (GeneTex S, Sino Biologics S, and Sino Biologics N), positive signals were observed in all TMAs ranging between 6 and 17% depending on the antibody (Fig. 4 *C* and *D* and Dataset S3). Positive cells were most commonly epithelium but also, macrophages; positive signals were seen in a variety of tissues. In all IHC studies, appropriate negative and positive controls were stained simultaneously; positive controls from COVID-19 lung autopsy tissue demonstrated focal staining of alveolar pneumocytes and macrophages (*SI Appendix*, Fig. S4*B*). These data suggest that SARS-CoV-2–targeted antibodies may stain some tissues nonspecifically, or rather, they may target unidentified human tissue antigens. Poor specificity of SARS-CoV-2–targeted antibodies with IHC (53%) has been noted elsewhere in normal tissues when compared with ISH (100%) (18).

## Discussion

In this detailed study of a cohort of patients with chilblain eruptions presumed to be caused by SARS-CoV-2, we do not find evidence supporting an association with prior infection. Many observational reports have offered circumstantial evidence pointing toward an association with SARS-CoV-2 infection, although the immunological and PCR data have been more limited, with only the minority of cases having evidence of infection (1, 4–8) and some studies having none whatsoever (2, 3, 12). Importantly, PC cases are being diagnosed and managed differently than idiopathic chilblains due to this presumed relationship with SARS-CoV-2 (19). In this study, we provide compelling evidence of prior SARS-CoV-2 infection in our cohort with a temporal association in local infection rate (Fig. 1*B*) and the co-occurrence of cases from the same household (Fig. 1*C*) as well as a substantial fraction reporting exposure (35%) to infected individuals and symptoms indicative of infection (35%) (Fig. 1*D*). However, only 2 of the 21 patients (9.5%) with chilblain eruptions had evidence of a prior SARS-CoV-2 infection. None of the cases within households had evidence of infection. This number (9.5%) approximates regional seroprevalence of SARS-CoV-2 at the time (8.5%) (20) in Connecticut, indicating that that this outcome could have been expected by chance from an unselected population of Connecticut residents during this period.

Recently, a study examining PCR, antibody, and T cell responses in active chilblain eruptions was published (21). All PCR studies were negative, and only 4 of 50 samples had a positive antibody response at the two tested time points. Their study demonstrated T cell responses to SARS-CoV-2 antigens that were comparable with healthy controls. These data are complementary to our study in that they fail to show evidence of infection in an early presumed exposure period, whereas our study describes similar results in the convalescent state. Altogether, these studies better support an alternative explanation for an increase in the incidence of chilblains, possibly attributed to altered behavior during the pandemic (e.g., not wearing socks/shoes at home during quarantine) and/or increased awareness due to reports of covid toe in the media as has been proposed elsewhere (16, 22). In support of this theory, multiple initial encounters in our cohort documented the patient’s concern for a covid toe in the history of present illness, confirming the increased awareness of this entity by patients at the time.

An additional potential explanation for the lack of antigen-specific responses seen in these patients is a robust mucosal innate immune response that may prevent the adequate sampling of antigen to develop detectable T cell and antibody responses. Similarly, seronegative abortive infections have recently been described in SARS-CoV-2, which are associated with an innate immune signature and cross-protective T-cell reactivity to SARS-CoV-2 replication-transcription complex; whether this can explain some aspects of the association between SARS-CoV-2 and PC remains unstudied (23). It is reasonable to hypothesize that PC patients may develop chilblains as a consequence of their potent type I interferon response, which is a clinical finding also seen in interferonopathies like Aicardi–Goutières syndrome. An intriguing study exploring this hypothesis showed strong type I interferon responses with ex vivo stimulation of whole blood with α-CD3 and TLR7/8 agonists in PC patients (24). However, the lack of an age-matched healthy control group confounds the interpretation of these results given the well-studied age-dependent decrease in type I interferon responses (25–29). Presumably, PC patients would have higher type I interferon responses than age-matched healthy controls if this hypothesis is indeed true; however, this was not studied here. Additionally, how comparable ex vivo stimulation of whole blood in the above conditions would be to the cellular milieu at the mucosal site of initial infection with actual virus is an ongoing question.

This hypothesized relationship between innate type I interferon responses potentially driving PC does merit further discussion. PC has been described as a “late” finding in response to COVID-19, occurring typically after symptom onset in a range from 1 to 4 wk (1, 4, 19). In mild cases of SARS-CoV-2 infection in younger, healthier patients—the described population primarily affected by PC—both viral load and type I interferon responses peak in the first week and decline thereafter (30, 31). Accordingly, there is a paucity of evidence to indicate that at the time patients develop PC, they would truly have systemic elevation in type I interferons and interferon-stimulated genes. Elevated interferon signatures are observed in PC skin biopsies (32), although these are not specific to an etiology and are characteristic of gene expression profiles seen in autoimmune skin diseases, like lupus and dermatomyositis, which are not driven by viral infection. One additional counterpoint to this suspected relationship is that while some interferonopathy patients do feature acral chilblain-like eruptions, these eruptions are not linked to systemic treatments with interferon-α or -β for various diseases, like hepatitis and cancer (33–35). Thus, systemic elevation in type I interferons per se is unlikely sufficient to drive PC.

The positive staining of S in the eccrine gland and endothelium of PC biopsies has been used as additional evidence supporting prior infection (10, 13). The reanalysis of our previously published cases raises questions about the specificity of staining for SARS-CoV-2 antigens. In one of the cases from our original series, we demonstrate that this patient (PC #23) lacks evidence of prior infection with serology studies and TCR sequencing. We additionally show through multiple stains a variable pattern of positivity; while our previous IHC identified positive staining with one S antibody, we subsequently found negative staining with two other antibodies (S and N) and a lack of detectable RNA (Fig. 4*B*). Finally, our staining of prepandemic TMAs suggests that these antibodies may stain tissues nonspecifically or possibly stain other host antigens. The possibility of SARS-CoV-2–targeted antibodies binding host antigens has been raised in previous studies showing S- and Nucleoprotein-targeted monoclonal antibodies specifically binding an array of broadly expressed human proteins (36, 37).

Electron microscopy has similarly been used to support SARS-CoV-2 in PC biopsies (10); however, the high interobserver variability associated with this technique has drawn criticism and arguments that so-called viral particles are in fact normal subcellular organelles (38, 39). In addition to this question, one additional factor that has not been raised is how, even if previously infected, the virus would end up in the toes of patients who generally have no other symptoms; previous studies have demonstrated comparable patient groups with mild or asymptomatic SARS-CoV-2 infection lacking detectable virus in the blood (40, 41), the presumed route by which virus would reach this distal site. A related question that has not been adequately addressed is that if S antigen is indeed lodged in the toe of PC patients, why do they not develop antigen-specific antibodies given that S is the immunodominant SARS-CoV-2 antigen and used as the basis for all major SARS-CoV-2 vaccines to generate robust antibody responses (42–46)? These important questions, when considered alongside our findings, should prompt additional critical review of existing PC Spike IHC data. Given increasing reports indicating a lack of prior infection, the likelihood of this being bona fide staining becomes more remote.

Our study has limitations in that it is a single-center small cohort study. We have been unable to acquire other PC tissue samples that previously stained positive for SARS-CoV-2 antigens, rendering our observations isolated to our own small collection, which may not be representative of those published elsewhere. On balance, our studies do not support an association between PC and SARS-CoV-2 infection. More studies are indicated to further understand this connection.

## Methods

### Patients.

The clinical study of this cohort of patients and retrospective analysis of skin biopsies were approved by the Yale Human Research Protection Program Institutional Review Board (protocol identification nos. 2000027690 and 2000022585, respectively). All participants provided informed consent at the time of enrollment into the study.

### Isolation of Plasma and PBMCs.

Whole blood was collected in sodium heparin-coated blood vacutainers (BD) and kept on gentle agitation until processing. Plasma samples were collected after centrifugation of whole blood at 600 × *g* for 20 min at room temperature (RT) without brake. The undiluted plasma was transferred to 15-mL polypropylene conical tubes, aliquoted, and stored at −80 °C. The PBMC layer was isolated according to the manufacturer’s instructions using SepMate tubes (StemCell). Cells were washed twice with phosphate-buffered saline (PBS) before counting. Pelleted cells were briefly treated with ACK lysis buffer (ThermoFisher) for 2 min and then counted. Percentage viability was estimated using standard Trypan blue staining and an automated cell counter (ThermoFisher). PBMCs were stored at −80 °C for downstream studies.

### SARS-CoV-2 ELISA Antibody Studies.

Serological ELISAs were carried out as previously shown (47, 48) with minor adjustments. High-binding 1,536-well plates (Perkin-Elmer; SpectraPlate 1536 HB) were coated with 3 µL of 1 µg/mL SARS-CoV-2 S ectodomain, RBD, or N protein in PBS using Fritz Gyger Certus Flex, incubated at 37 °C for 1 h in a ThermoFisher rotating plate incubator, and washed three times with PBS 0.1% Tween-20 (PBS-T) using Biotek El406. Plates were blocked with 10 µL of 5% milk in PBS-T for 1.5 h using Biotek Multiflo FX peristaltic dispensing technology. Samples inactivated with 1% Triton X-100 and 1% tributyl phosphate were diluted in sample buffer (1% milk in PBS-T), and a serial dilution (range: 0.02 to 1.6 × 10^−4^) was carried out (volume: 3 µL per well) on an ECHO 555 acoustic dispenser (Labcyte) using contactless ultrasound nanodispensing. After the sample incubation for 2 h at RT, the wells were washed five times with wash buffer, and the presence of anti–SARS-CoV-2 antibodies was detected using horseradish peroxidase (HRP)-linked anti-human IgG antibody (Peroxidase AffiniPure Goat Anti-Human IgG, Fcγ Fragment Specific; Jackson; 109-035-098 at 1:4,000 dilution), HRP-linked anti-human IgA antibody (Goat Anti-Human IgA Heavy Chain Secondary Antibody, HRP; ThermoFisher Scientific; 31417 at 1:750 dilution), and HRP-linked anti-human IgM antibody (anti-human IgM μ-chain–specific antibody; Sigma-Aldrich; A6907 at 1:3,000 dilution), all of them diluted in sample buffer at 3 µL per well dispensed on Biotek Multiflo FX. The incubation of the secondary antibody for 1 h at RT was followed by three washes with PBS-T, the addition of 3 µL per well of Tetramethylbenzidine (TMB) substrate solution with a Biotek Multiflo FX syringe dispenser, incubation of 3 min at RT, and the addition of 3 µL per well 0.5 M H_2_SO_4_ using Fritz Gyger Certus Flex. The plates were centrifuged in the Agilent automated microplate centrifuge after all dispensing steps, except for the addition of TMB. The absorbance at 450 nm was measured in a plate reader (Perkin-Elmer; EnVision), and the inflection points of the sigmoidal binding curves [i.e., the p(EC_50_) values of the respective sample dilution; p(EC50) is the negative logarithm of one-half the maximal concentration (EC50)] were determined using a custom-designed fitting algorithm (47), with plateau and baseline inferred from the respective positive and negative controls in a platewise manner. Negative p(EC_50_) values, reflecting nonreactive samples, were rescaled as zero.

### SARS-CoV-2 SERA Antibody Studies.

Patient serum was incubated with the fully random 12-mer bacterial display peptide library (1 × 10^10^ diversity, 10-fold oversampled) at a 1:25 dilution in a 96-well deep-well plate format. Antibody-bound bacterial clones were selected with 50 µL Protein A/G Sera-Mag SpeedBeads (GE Life Sciences) or by incubation with a biotinylated anti-human IgM antibody (Jackson ImmunoResearch; 1:100 dilution) followed by a second incubation with 100 μL Dynabead MyOne Streptavidin T1 conjugated magnetic beads (ThermoFisher; 65602). The selected bacterial pools were resuspended in growth media and incubated at 37 °C with shaking overnight at 300 rpm to propagate the bacteria. Plasmid purification, PCR amplification of peptide encoding DNA, and barcoding with Illumina well-specific PCR indices were performed as previously described (49). Samples were normalized to a final concentration of 4 nM, pooled (94 samples per sequencing run), and sequenced on the Illumina NextSeq500. A detailed description of the SERA technology is described elsewhere (49).

### In Vitro T Cell Simulation Studies.

Cells were thawed and plated at concentrations of 0.5 to 1 × 10^6^ in 200 µL of Roswell Park Memorial Institute (RPMI) growth medium 1640 (Gibco) supplemented with 1% sodium pyruvate (NEAA), 100 U/mL penicillin/streptomycin (Gibco), and 10% fetal bovine serum at 37 °C and 5% CO_2_. PBMCs were then stimulated with S peptide pools in culture over the course of 7 d. On day 1, cells were washed and stimulated with PepMix SARS-CoV-2 S glycoprotein pool 1 and pool 2 (GenScript) at a final concentration of 1 µg/mL per peptide; stimulation controls received PBS. Cells were incubated for 6 d with a single media change performed on day 4. On day 6, cells were restimulated with 10 µg/mL per peptide for 12 h, with the last 6-h incubation performed in the presence of the protein transport inhibitor mixture (ThermoFisher; 1:500 dilution). Following this incubation, cells were washed with PBS 2 mM ethylenediaminetetraacetic acid and prepared for analysis by flow cytometry.

### REAP.

REAP technology offers high-throughput profiling of autoantibodies that bind to conformational epitopes and was developed in A.M.R.’s laboratory at Yale. The REAP studies in this manuscript were performed in collaboration with his laboratory. Briefly, genetically barcoded yeast libraries expressing antigens of interest are incubated with patient serum. IgG-coated yeast is then isolated magnetically, and bound antigens are identified by deep sequencing to associate specific barcodes and the original antigen of interest. REAP scores were then calculated based on aggregate and clonal enrichments; a more detailed discussion of the technology and protocols is described elsewhere (15).

### Flow Cytometry.

Antibodies used for the peptide stimulation experiment are as follows: BB515 anti–hHLA-DR (G46-6; 1:400; BD Biosciences), BV605 anti-hCD3 (UCHT1; 1:300; BioLegend), BV785 anti-hCD4 (SK3; 1:200; BioLegend), APCFire750 or BV711 anti-hCD8 (SK1; 1:200; BioLegend), and BV711 anti-hCD38 (HIT2; 1:200; BioLegend). After finishing the stimulation described above, cells were resuspended in Live/Dead Fixable Aqua (ThermoFisher) for 20 min at 4 °C. Following a wash, cells were blocked with Human TruStain FcX (BioLegend) for 10 min at RT. Antibody mixtures were added directly to cells for 30 min at RT. Prior to analysis, cells were washed and resuspended in 100 μL of 4% paraformaldehyde for 30 min at 4 °C. Following this incubation, cells were washed and prepared for analysis on an Attune NXT (ThermoFisher). Data were analyzed using FlowJo software version 10.6 software (Tree Star). Our gating strategy for flow cytometry experiments is seen in *SI Appendix*, Fig. S3.

### T Cell Receptor Sequencing.

Sequencing of CD8 TCRβ CRD3 regions from patient samples was performed using the immunoSEQ assay (Adaptive Biotechnologies). Genomic DNA was isolated from PBMC samples using a kit (Qiagen) and amplified in a bias-controlled multiplex PCR followed by high-throughput sequencing (performed at the Yale Center for Genome Analysis). Sequencing filtering and quantitation were performed as previously described (50). TCR sequences were mapped against a set of TCR sequences that are known to react to SARS-CoV-2 by Multiplex Identification of T-Cell Receptor Antigen Specificity (51). TCRs that react were additionally screened for enrichment in COVID-19–positive repertoires collected as part of ImmuneCODE and compared with negative controls for filtering of repertoires that may be public or cross-reactive to common antigens. The clonal depth metric in Fig. 3 is calculated as the sum frequency of SARS-CoV-2 annotated rearrangements divided by the total number of unique productive rearrangements; for this calculation, only SARS-CoV-2 rearrangements with greater than two templates were included for quantitation (Fig. 3 *A* and *B*).

### IHC.

Staining of all samples was performed in the clinical Yale Dermatopathology laboratory. Primary antibodies used for IHC are as follows: Sino Biologics S (catalog no. 40150-T62-COV; 1:200 dilution/15-min incubation, low-pH antigen retrieval), Sino Biologics N (catalog no. 40143-R001; 1:1,000 dilution/30-min incubation, high-pH antigen retrieval), ThermoFisher N (catalog no. MA1-7404; 1:400 dilution/30-min incubation, low-pH antigen retrieval), and GeneTex S (catalog no. GTX632604; 1:400 dilution/30-min incubation, low-pH antigen retrieval). After deparrafinization, antigen retrieval was performed using either low- or high-pH retrieval buffer solutions from Leica. For antigen retrieval, solution was preheated to 85 °C. Slides are then placed in buffer, and the heat is increased to 95 °C for 30 min. Thereafter, slides were kept in solution to cool back down to 85 °C. Slides were then placed in RT buffer. TMAs were purchased from US Biomax (MN0961 and FDA999X) and confirmed to only include tissue prior to 2019. Dataset S3 has details on the grading and specific tissues for each TMA and antibody. For all experiments, isotype negative controls and positive control COVID-19 lung tissues were run in parallel.

### Real-Time PCR.

Real-time PCR was performed from formalin-fixed tissues using the Applied Biosystems combination TaqMan PCR kit targeting three regions of SARS-CoV-2, including ORF1ab, S, and N with RNase P as control. The RecoverAll nuclear acid isolation kit (ThermoFisher) was used to isolate RNA from formalin-fixed samples. TaqMan Fast Virus 1-Step Master Mix (ThermoFisher) was used to generate complementary DNA (cDNA). All samples were diluted to a concentration of 0.5 ng of input cDNA prior to PCR. Using tissues from a mouse model of SARS-CoV-2 (52), we confirmed detection of SARS-CoV-2 down to 1 pg of input cDNA. Real-Time PCR was performed on a BioRad CFX96 instrument. Cycling instructions were as follows (as indicated in the TaqMan Fast Virus 1-Step protocol): reverse transcription at 50 °C for 5 min and activation at 95 °C for 20 s followed by 40 cycles of denaturation (95 °C × 3 s) and anneal/extension (60 °C × 30 s).

### Statistics.

For multiple group comparisons, nonparametric Kruskal–Wallis one-way ANOVA with post hoc Dunn’s test was used in GraphPad Prism 8.0 software. Details of specific experiments can be found in the figures.

## Supplementary Material

Supplementary File

Supplementary File

Supplementary File

Supplementary File

## Data Availability

All study data are included in the article and/or supporting information.
