# Supplementary material for: Lack of association between pandemic chilblains and SARS-CoV-2 infection
Source: Proc Natl Acad Sci U S A. 2022 Feb 25;119(9):e2122090119. doi: 10.1073/pnas.2122090119 (PMC8892496; doi:10.1073/pnas.2122090119)
Supplement: Supplementary File [file pnas.2122090119.sapp.pdf]

## Supplementary Appendix

| Case Number | Age | Gender | Clinical | PCR | Antibody | Symptoms | Known or Likely Exposure | Contact with similar rash | Date of rash | Initial Labs | Date of Blood Draw |
|---|---|---|---|---|---|---|---|---|---|---|---|
| 1 | 12 | M | chillblain - toes | neg | neg | no | no | no | mid April | none | 8/4/2020 |
| 2 | 14 | F | chillblain - fingers, toes | NC | neg | yes | no | no | mid February | APLS, CA, CBC, cryo, SPEP | 8/6/2020 |
| 3 | 16 | F | chillblain - toes | neg | neg | no | yes | yes | mid April | CBC | 8/11/2020 |
| 4 | 16 | M | chillblain - toes | neg | neg | yes | no | yes | early May | ANA+ (1:320), neg dsDNA and Sm Ab; CBC | 8/11/2020 |
| 5 | 27 | M | chillblain - toes | neg | neg | no | no | no | mid April | none | 6/30/2020 |
| 6 | 27 | F | chillblain - fingers, toes | neg | neg | no | yes | no | late April | APLS, CA | 8/25/2020 |
| 7 | 32 | F | chillblain - toes | neg | NC | no | no | no | late May | none | 6/25/2020 |
| 8 | 37 | F | chillblain - toes | neg | NC | no | no | no | late July | ANA, CBC; known Hep C positive | 8/26/2020 |
| 9 | 39 | F | chillblain - toes | neg | NC | no | no | yes | late April | CBC | 7/14/2020 |
| 10 | 39 | F | chillblain - fingers | NC | NC | yes | no | no | late May | none | 8/4/2020 |
| 11 | 40 | M | chillblain - foot, toes | neg | NC | no | no | yes | late March | none | 7/14/2020 |
| 12 | 42 | F | exanthem | pos | pos | yes | yes | no | early April | CBC | 8/25/2020 |
| 13 | 42 | F | chillblain - toes | NC | NC | no | no | no | early May | none | 8/4/2020 |
| 14 | 46 | M | chillblain - fingers, knee, foot, toes | neg | neg | yes | yes | no | mid Apirl | none | 6/30/2020 |
| 15 | 47 | F | unilateral extremity livedo | NC | NC | yes | yes | yes | mid April | CBC w/ moderate neutropenia and reactive lymphocytes | 6/30/2020 |
| 16 | 47 | F | chillblain - toes | pos | NC | yes | yes | no | April | CBC | 8/31/2020 |
| 17 | 50 | M | chillblain - toes | neg | NC | yes | no | no | April | CBC | 6/30/2020 |
| 18 | 50 | F | chillblain - toes | neg | neg | no | yes | yes | early May | ANA+ (1:40); CBC | 8/4/2020 |
| 19 | 56 | M | chillblain - fingers, toes* | neg | neg | no | yes | yes | late April | none | 8/4/2020 |
| 20 | 56 | F | chillblain - toes | neg | neg | no | no | no | late April | low + IgM Phosphatidylserine Ab, low + IgM Cardiolipin Ab; ANA | 8/4/2020 |
| 21 | 61 | F | chillblain - toes | NC | NC | no | no | no | late April | none | 8/4/2020 |
| 22 | 61 | F | acral/extremity chillblain* | NC | neg | no | no | no | early May | CBC w/ leukopenia; ANA, APLS | 8/4/2020 |
| 23 | 82 | F | chillblain - fingers* | neg | neg | no | no | no | March | CBC | 8/13/2020 |

**Table S1: Expanded clinical information on PC cohort.** Definitions of clinical survey questions and reporting are described individually below. **Symptoms**: defined as preceding 2 months with symptoms suggestive of COVID-19 infection including fever, cough, SOB, recent change in sense of smell or taste. **Exposure:** defined as preceding 2 months exposure to someone with symptoms attributable to COVID-19 or a person with known positive covid test. **Contact with similar rash**: defined as preceding 2 months with exposure to someone with a rash attributed to COVID-19 infection (positive instances were chilblains or livedo reticularis). **Exclusion criteria**: history of chilblains or cutaneous lupus erythematosus. Values listed in red in the Initial Labs column indicate positivity/abnormality; otherwise text in black indicates normal values. *biopsy performed; histopathology consistent with chilblains including dermal edema and perivascular lymphocytic inflammation. Additional ANA testing was performed on 22 samples (all except PC #16) using ANA Kit (Antibodies Incorporated) with sera diluted 1:160; no new positive ANA values were observed. Ab = antibody; ANA = antinuclear antibody;  APLS = anti phospholipid syndrome; CA = cold agglutinin; CBC = complete

blood count; cryo = cryoglobulins; dsDNA = double stranded DNA; Sm = Smith; SPEP = serum protein electrophoresis.

| | COVID Diagnosis Date | Time since infection | COVID Diagnosed By | Hospitalized For COVID | RBD ELISA (cut off 0.28) | SERA IgG (cut off 25) | SERA IgM (cut off 25) |
|---|---|---|---|---|---|---|---|
| MC 1 | 12/22/2020 | 4 months post infection | Blood test (antibody test) | No | 0.53 | 34.7 | 10.7 |
| MC 2 | 5/28/2020 | 1 year post infection | Nasal swab (PCR/RNA/NAT) | No | 1.45 | 58.9 | 14.2 |
| MC 3 | 3/13/2020 | 1.5 month postsymptom onset | PCR | No | 1.080 | 61.2 | 128.6 |
| | 3/13/2020 | 4 months postsymptom onset | PCR | No | 1.088 | 40.7 | 68.2 |
| | 3/13/2020 | 6 months postsymptom onset | PCR | No | 0.983 | 49.2 | 48.8 |
| | 3/13/2020 | 9 months postsymptom onset | PCR | No | 0.588 | 34.8 | 58.7 |

**Table S2: Additional SERA positive controls.** Three different individuals with mild prior SARS-CoV-2 infection (mild covid, or MC) demonstrating that ELISA and SERA assays can detect prior infection for up to a year after initial diagnosis. MC #3 has four different time points collected starting from 1.5 months to 9 months after diagnosis. NAT = nucleic acid amplification test; PCR = polymerase chain reaction; RBD = receptor binding domain; SERA = serum epitope repertoire analysis.

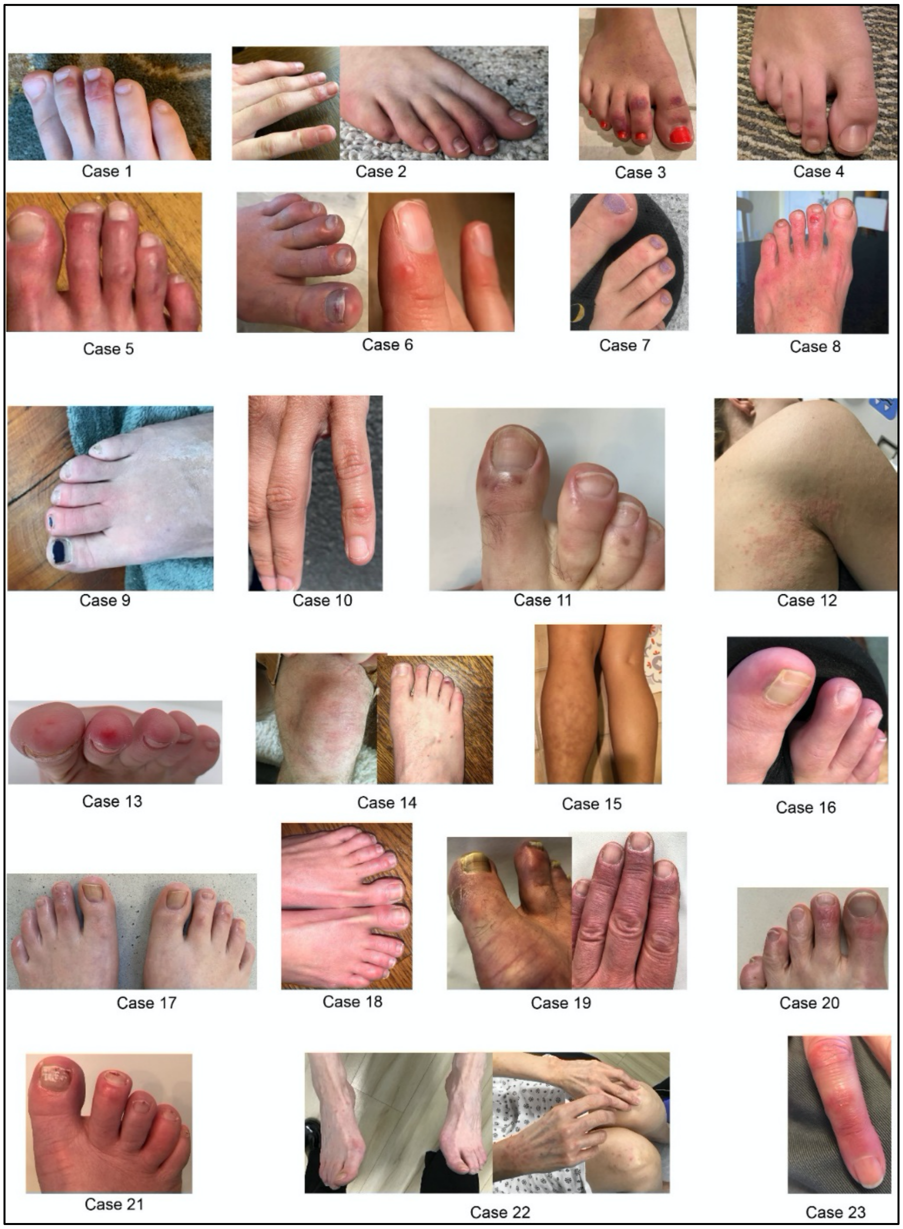


**Figure S1: Clinical photographs of all cases included in PC cohort.** 21 of 23 cases were considered chilblains incorporating both clinical and histopathologic studies (when available). Case 12 was an exanthem in the setting of acute COVID-19 disease (patient was hospitalized). Case 15 was unilateral livedo reticularis. All classes of eruptions have a published association with SARS-CoV-2.

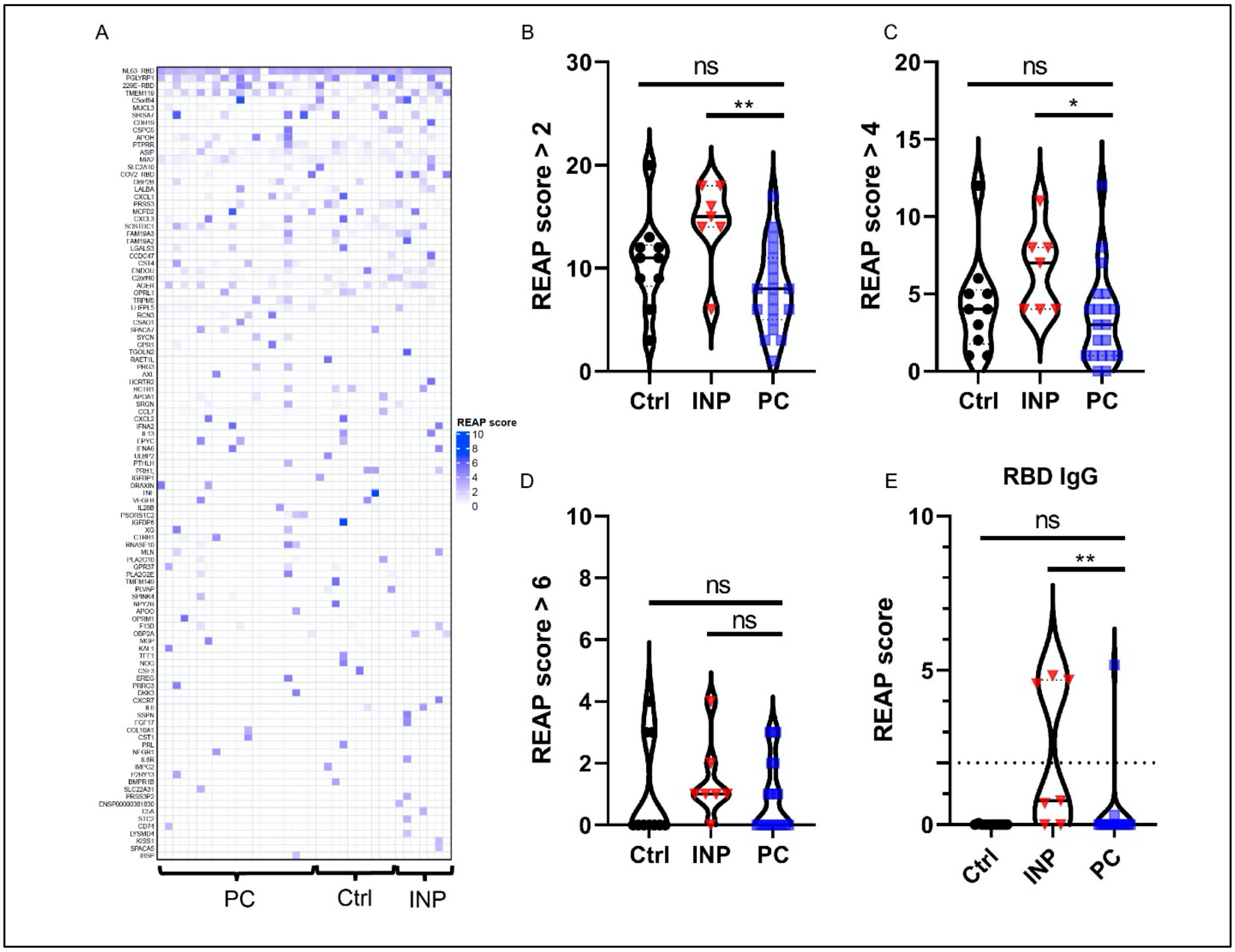


**Figure S2: REAP analysis of PC cohort compared to controls.** A) Heatmap of REAP IgG results comparing 22 PC cases, 10 healthy controls, and 7 moderate severity inpatients. B, C, and D) Violin plots of PC cases (22) compared to Ctrl (10) and INP (7) cases. For visualization purposes cases with values 3 SD above the median were removed, resulting in removal of 1 PC case in B. E) REAP testing of RBD antigen; no new positives were identified in PC cohort. For all experiments with significance testing, Kruskal-Wallis test with post-hoc Dunn's test for multiple comparisons was employed. Ctrl = no exposure negative controls; INP = moderate severity inpatients; PC

= pandemic chilblains; REAP = rapid extracellular antigen profiling; SD = standard deviation. ns, not significant; *, $P < .05$; **, $P < .01$.

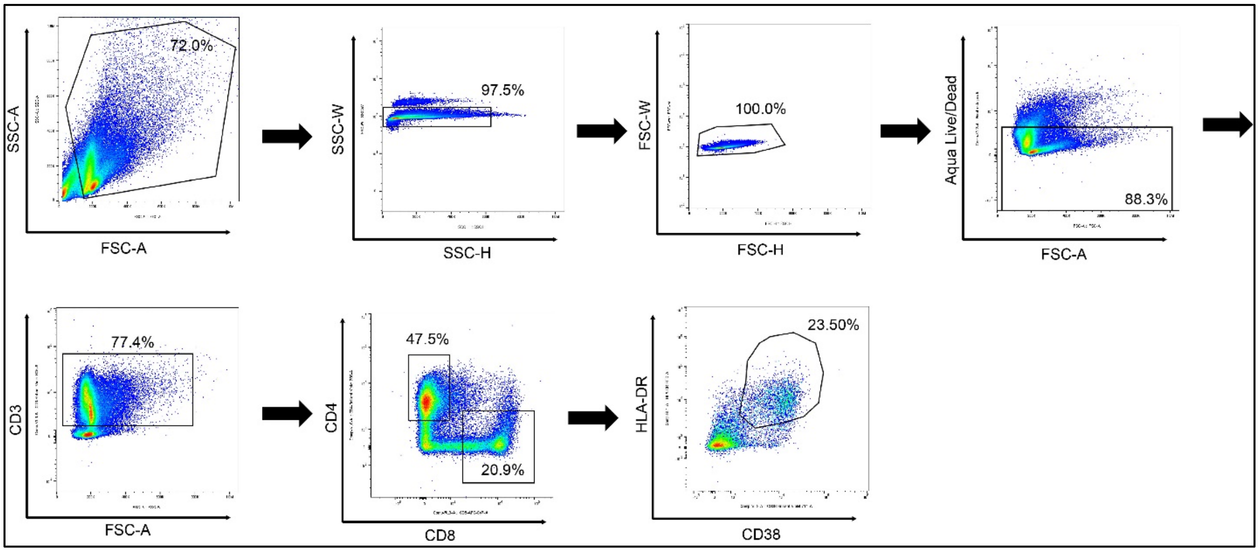


**Figure S3: Flow cytometry gating strategy.** Please see methods for detailed description of peptide stimulation and flow cytometry protocol.

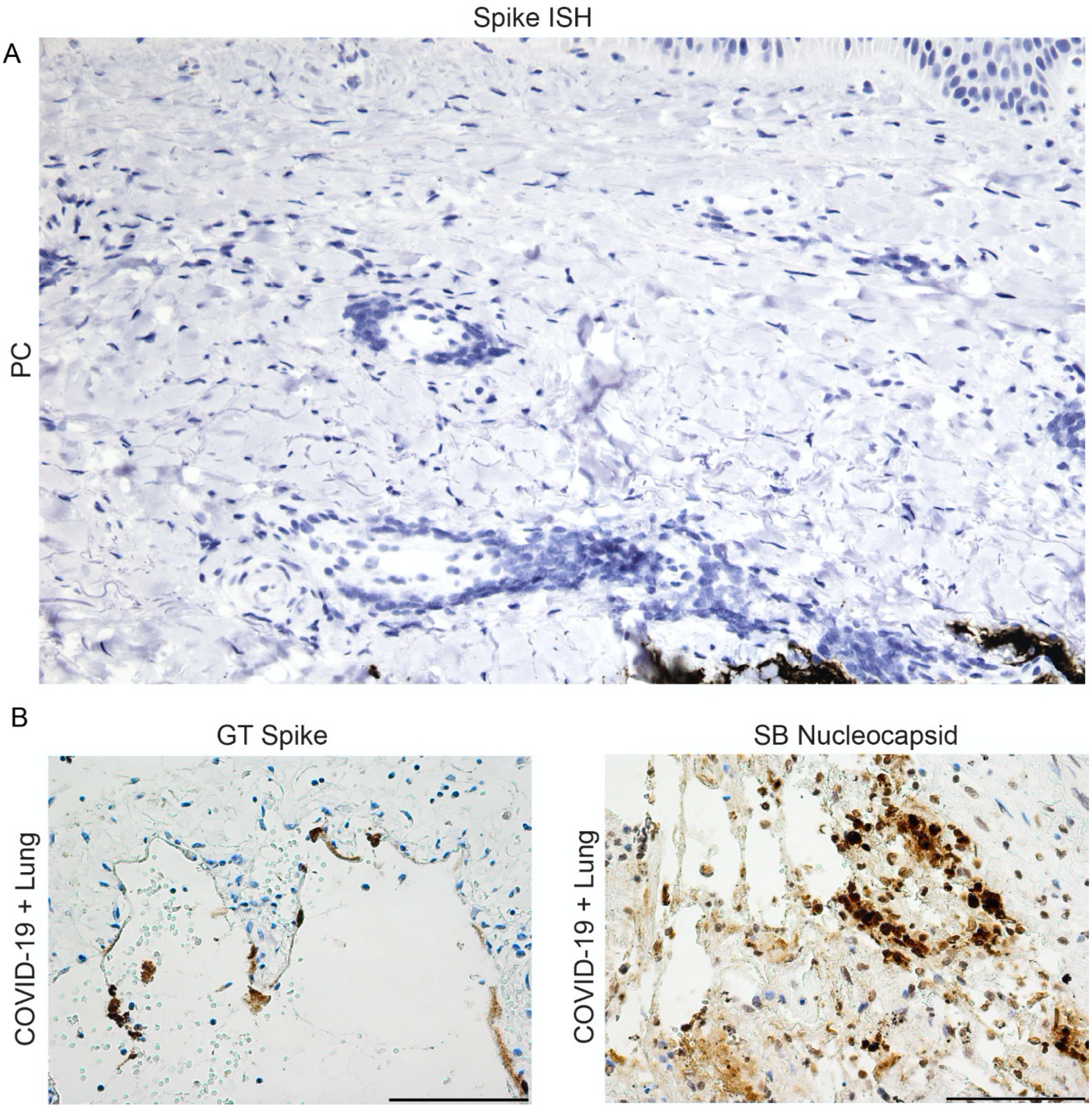


**Figure S4: Additional histopathology ISH and IHC staining controls for PC and COVID-19 lung tissues.** A) PC tissue stained with Spike targeted RNA in situ hybridization (ISH) probe indicating no Spike RNA present in the tissue. Contrast with the images shown in Figure 4A with staining of Spike in the endothelium and eccrine glands. B) Positive control COVID-19 lung tissues stained with the indicated antibodies;

focal staining with Spike and Nucleocapsid was observed in alveolar pneumocytes and macrophages. GT = GeneTex; SB, Sino Biologics; Scale bars = 100 uM; Image A is 200x original magnification, images in B are 400x.

**Yale IMPACT Author List**

Abeer Obaid, Alice Lu-Culligan, Allison Nelson, Anderson Brito, Angela Nunez, Anjelica Martin, Annie Watkins, Bertie Geng, Chaney Kalinich, Christina Harden, Codruta Todeasa, Cole Jensen, Daniel Kim, David McDonald, Denise Shepard, Edward Courchaine, Elizabeth B. White, Eric Song, Erin Silva, Eriko Kudo, Giuseppe DeIuliis, Harold Rahming, Hong-Jai Park, Irene Matos, Jessica Nouws, Jordan Valdez, Joseph Fauver, Joseph Lim, Kadi-Ann Rose, Kelly Anastasio, Kristina Brower, Laura Glick, Lokesh Sharma, Lorenzo Sewanan, Lynda Knaggs, Maksym Minasyan, Maria Batsu, Mary Petrone, Maxine Kuang, Maura Nakahata, Melissa Campbell, Melissa Linehan, Michael H. Askenase, Michael Simonov, Mikhail Smolgovsky, Nicole Sonnert, Nida Naushad, Pavithra Vijayakumar, Rick Martinello, Rupak Datta, Ryan Handoko, Santos Bermejo, Sarah Prophet, Sean Bickerton, Sofia Velazquez, Tara Alpert, Tyler Rice, William Khoury-Hanold, Xiaohua Peng, Yexin Yang, Yiyun Cao, and Yvette Strong
